# CD4 T cell contact drives macrophage cell cycle G0-G1 transition

**DOI:** 10.1038/s41392-024-02053-2

**Published:** 2024-12-13

**Authors:** Petra Mlcochova, Na Zhao, Omar Shabana, Roman Fischer, Ravindra K. Gupta

**Affiliations:** 1 Cambridge Institute of Therapeutic Immunology & Infectious Disease (CITIID), University of Cambridge, Cambridge, UK; 2 University of Oxford, Oxford, UK

**Keywords:** Infection, Senescence

**Dear Editor**

Macrophages are cells implicated in a diverse array of functions such as defence against pathogens, tissue repair /homoeostasis, and anti-cancer activity.^1,2^ Macrophages are also targets of virus infection, and SAMHD1 is known to restrict lentiviruses through depletion of intracellular dNTP and inhibition of reverse transcription. We previously showed that HIV-1 bypasses this restriction by infecting macrophages that have entered the cell cycle and deactivated SAMHD1 antiviral activity through CDK1 mediated phosphorylation of SAMHD1 at T592.^3^

Macrophages form ‘immunological synapses’ with CD4 T cells during MHC II dependent antigen presentation. These involve contact between the two cells via a number of interacting surface proteins including LFA-1 and ICAM-1 in addition to peptide loaded MHC interaction with the cognate TCR on CD4 T cells. Much less is known regarding antigen-independent contact between macrophages and T cells. Here we show that contact leads to cell cycle progression in macrophages.

Monocyte derived macrophages were co-cultured with autologous CD4 + T cells, as well as under control conditions of media alone (M0), or stimulated with LPS/IFNgamma to induce M1-like macrophages or IL4/IL-13 to induce M2-like polarised macrophages. Macrophages were then washed to remove CD4 T cells and (i) infected with VSV-G pseudotyped HIV-1 lentivirus (PV), or (ii) stained for markers of cell cycle progression.

Macrophages co-cultured with CD4 T cells were an order of magnitude more susceptible to infection compared to M0 macrophages, co-incident with increased expression of the marker for G0-G1 transition, MCM2 (minichromosome maintenance protein 2)^3^ (Fig. 1a). MCM2 is a nuclear protein expressed from G1 through to mitosis and absent from cells in G0 phase.^4^ Western blot analysis confirmed higher cellular expression of MCM2 under co-culture conditions and also demonstrated increased CDK1 expression and SAMHD1 phosphorylation with little change in overall SAMDH1 expression (Fig. 1a), consistent with changes that are known to be associated with G0-G1 transitions in MDM.^3^

**Fig. 1:**
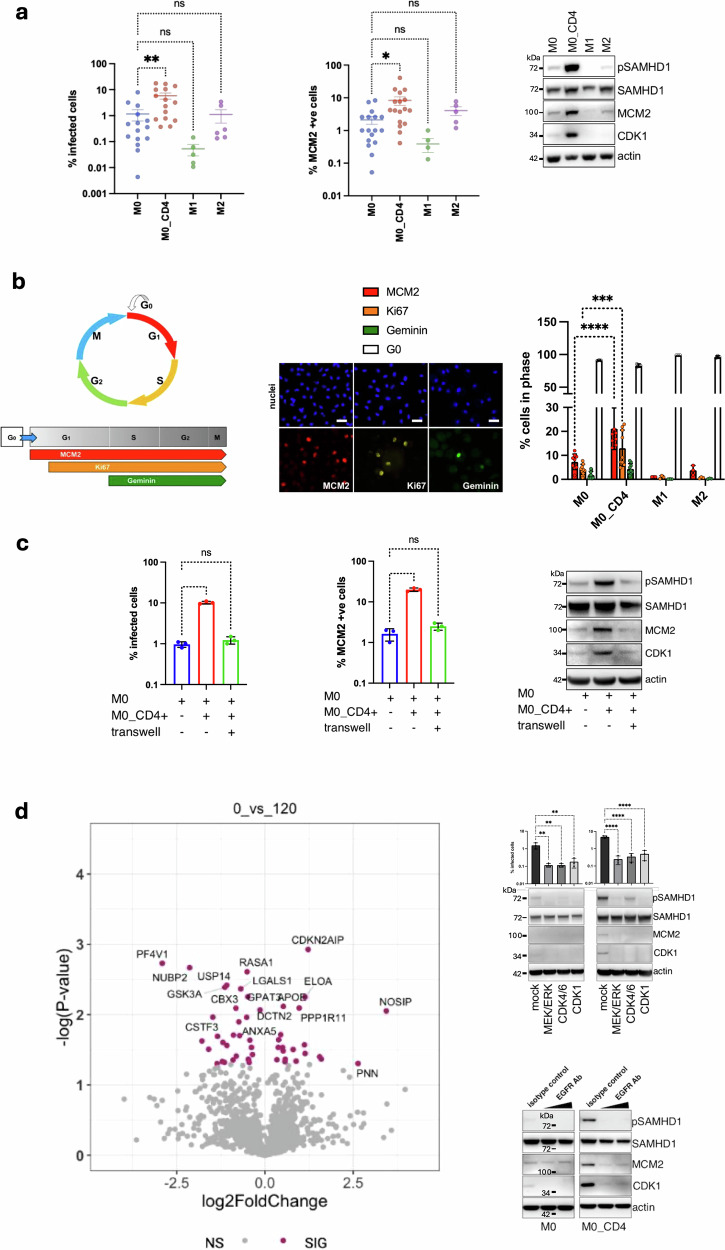
Direct contact with CD4 + T cells drives EGFR dependent G0-G1 transition in macrophages **a** Differentiated macrophages were co-cultured with activated autologous CD4 + T-cells, or stimulated by LPS/Interferon gamma (to polarize cells into M1 macrophages), or IL-4/IL-13 (into M2 macrophages). CD4 + T-cells were washed off 2 days later and cell cycle, viral permissivity was analysed. **a** Macrophages were infected with VSV-G pseudotyped HIV-1 expressing GFP (left panel). Cells were fixed 2 days post-infection and percentage of GFP positive cells was quantified by using automated microscopic platform. Graphs represent average of *n* = 15 (M0, M0_CD4); *n* = 6 (M1-like, M2-like) biological replicates. Statistical analysis was performed using one-way ANOVA with Dunnett’s multiple comparisons test. ns, non-significant; ^∗^*p* < 0.1; ^∗∗^*p* < 0.01. Bars indicate mean with SD. Middle panel: Macrophages were fixed and stained for MCM2 (a marker of cell cycle entry) and percentage of positive cells was determined by using automated microscopic platform. Graphs represent average of *n* = 16 (M0, M0_CD4); *n* = 4 (M1-like, M2-like) biological replicates. Statistical analysis was performed using one-way ANOVA with Dunnett’s multiple comparisons test. ns, non-significant; ^∗^*p* < 0.1. Bars indicate mean with SD. Right panel Macrophages were lysed and cell lysates from a representative donor were used for immunoblotting using antibodies against CDK1, MCM2, SAMHD1 and pSAMHD1 T592. **b** A diagram of cell cycle depicting an expression of several cell cycle markers. None of the markers are present in G0; MCM2 is expressed in G1, S, G2,M; Ki67 is expressed in late G1, S, G2,M; geminin is expressed in S, G2,M. Macrophages following cell-cell contact (M0_CD4) were fixed and stained for cell cycle markers. A representative acquisition field from microscopic platform is shown in middle panel. Scale bars: 40 μm. Right panel shows Percentage of positive cells. At least 10^4^ cells were acquired and used for analysis. *n* = 3 biological replicates; Ordinary two-way ANOVA with Sidak’s multiple comparisons test: ^∗∗∗∗^*p* < 0.0001. Bars indicate mean with SD. **c**. Macrophages were plated at the bottom of transwell, CD4 + T-cells were added directly to macrophages to allow direct cell-cell contact or added in the top of transwell to prevent direct contact. CD4 + T-cell were washed off 2 days later, infected (HIV-1 GFP, left panel) or stained for MCM2 (middle panel). The percentage of GFP or MCM2 positive cells was quantified by using an automated microscopic platform. *n* = 3 biological replicates. Statistical analysis was performed using one-way ANOVA with Dunnett’s multiple comparisons test. ns, non-significant; ^∗∗∗∗^*p* < 0.0001. Bars indicate mean with SD. Right panel: Macrophages were lysed and cell lysates from a representative donor were used for immunoblotting with antibodies to CDK1, MCM2, SAMHD1 and pSAMHD1 T592. **d** left panel: Volcano plot showing differentially expressed phosphoproteins in macrophages following CD4 + T cell contact from 0 vs. 120 min versus the absence of T cell contact. Coloured proteins were significantly up or down regulated with a significance level of 0.1; right upper panel. Macrophages were untreated (M0) or co-cultured with CD4 + T-cells for 2 days in the presence or absence (mock) of inhibitors. T-cells were washed off and macrophages infected with VSV-G pseudotyped HIV-1 expressing GFP. Percentage of GFP positive cells was quantified 2 days later. Macrophages were lysed and cell lysates from a representative donor were used for immunoblotting. CDK1, MCM2 and SAMHD1 phosphorylation are markers of cell-cycle re-entry. *n* = 3 biological replicates. Statistical analysis was performed using one-way ANOVA with Dunnett’s multiple comparisons test. ^∗∗^*p* < 0.01; ^∗∗∗∗^*p* < 0.0001. Bars indicate mean with SD. right bottom panel Macrophages were untreated (M0) or co-cultured with CD4 + T-cells for 2 days in the presence of control isotype IgG antibody or increasing concentration of anti-EGFR antibody. T-cells were washed off and macrophages lysed. Cell lysates from a representative donor were used for immunoblotting

We next performed detailed analysis of cell cycle status to quantify cell cycle progression markers Geminin, MCM2 and Ki67 (Fig. 1b) by flow cytometry. G0 is quantified as cells with the absence of MCM2, Ki67, and Geminin. MDM cultured in human serum showed only modest MCM2 and Ki67 expression, consistent with our previous data^3^ (Fig. 1b). By contrast, macrophages co-cultured with CD4 T cells demonstrated significantly higher expression of MCM2 and Ki67 compared to M0 macrophages, indicating G0-G1 progression following co-culture.^3^

We next sought to establish whether direct cell-cell contact was required for CD4 T cells to induce G0-G1 progression in macrophages. Using a transwell system (Fig. 1c) we observed that placement of a barrier between macrophages and T cells abrogated the increased susceptibility to infection with VSG pseudotyped HIV-1 particles, as well as the cell cycle G0-G1 transition (Fig. 1c). Immunoblot confirmed that the transwell prevented an increase in MCM2, CDK1 and pSAMHD1 protein levels following the addition of CD4 T cells (Fig. 1c). We conclude that direct cell contact is required for T cells to induce G0-G1 transition and relieve SAMHD1-mediated viral restriction in macrophages.

In order to elucidate pathways that might be involved in mediating CD4 T cell contact dependent G0-G1 transition in macrophages we performed phosphoproteomic analysis. A number of phosphoproteins were either upregulated or downregulated. Frequently altered phosphoproteins included MAPKs, GSK3a, RASA1, CDKN2AIP and CDC42 that are known to be involved in the regulation of signal transduction and cell proliferation pathways (Fig. 1d). Given this result, activation of signal transduction and our previous work recognising the importance of the MEK/ERK pathway in the activation of macrophage G0-G1 transitioning,^3^ we hypothesised that MEK/ERK pathway is involved in CD4 T cell contact dependent cell cycle progression in macrophages. To test whether this pathway was responsible for the cell contact phenotype we used inhibitors of MEK (U0126), CDK4/6 (Palbociclib) and CDK1 (RO-3306). Each of the inhibitors almost completely inhibited MCM2, CDK1 and pSAMHD1 expression following T cell - macrophage contact, indicative of cell cycle arrest in G0 (Fig. 1d). This was reflected in concomitant blockade of infection with VSG pseudotyped HIV-1 particles following drug treatment, reminiscent of prior findings where genotoxic drugs induced macrophage cell cycle arrest^5^ (Fig. 1d).

Growth factors and mitogens use the canonical Ras-Raf/MEK/ERK signalling cascade to transmit signals from their receptors to regulate gene expression and cell cycle progression. EGFR (epidermal growth factor receptor) is one of the most important receptors in this pathway and is a well known cancer therapy target. We therefore treated macrophages with a monoclonal antibody to EGFR prior to co-culture with CD4 T cells. The antibody prevented G0-G1 transition even in the presence of CD4 T cells based on the expression of cell cycle associated markers MCM2 and CDK1, and pSAMHD1 (Fig. 1d). This suggests that EGFR signalling regulates cell cycle in macrophages following CD4 T cell contact.

These experiments demonstrating cell cycle progression following antigen-independent T cell contact represent a new phenotype. Phosphoproteomic analysis revealed that within 2 h of contact, cell cycle associated proteins were activated, and this was confirmed biochemically through inhibition of this pathway. We hypothesise that EGFR Ab blocks the interaction between EGFR on macrophages and ligands for EGFR on the T cell surface. Unlike exocrine secreted EGF, other EGFR ligands, including transforming growth factor-α, heparin-binding EGF (HB-EGF), and amphiregulin, are expressed by a variety of cells. Therefore, blockade of EGFR-ligand interactions at the macrophage surface would block downstream MEK/ERK signalling and changes in cell cycle.

Tissues such as lymph nodes and the genital tract are characterised by close cell contact between T cells and macrophages and are known HIV-1 reservoir sites. Our data suggest that T cell contact leads to deactivation of SAMHD1 antiviral activity following cell contact, and may contribute to observations that macrophages are a bona fide target for HIV-1. Similarly, other viruses infecting macrophages may be impacted by cell-cell contact and therefore blockade of cell-cell interaction may represent a future therapeutic opportunity in combating viral infections in myeloid cells.

## Supplementary information

supplementary material

## Data Availability

The mass spectrometry proteomics data have been deposited to the ProteomeXchange Consortium via the PRIDE partner repository (https://www.ebi.ac.uk/pride/) with the dataset identifier PXD048462 and 10.6019/PXD048462.
