# Correction: CD4 T cell contact drives macrophage cell cycle G0-G1 transition

**DOI:** 10.1038/s41392-024-02117-3

**Published:** 2025-01-06

**Authors:** Petra Mlcochova, Na Zhao, Omar Shabana, Roman Fischer, Ravindra K. Gupta

**Affiliations:** 1 Cambridge Institute of Therapeutic Immunology & Infectious Disease (CITIID), University of Cambridge, Cambridge, UK; 2 University of Oxford, Oxford, UK

**Keywords:** Infection, Senescence

Correction to: *Signal Transduction and Targeted Therapy* 10.1038/s41392-024-02053-2, published online 13 December 2024

In the original version of this article,^1^ the family names of second author Na Zhao was incorrectly published as “Zhou”, instead it should be “Zhao”.

The original article has been corrected.
