# Supplementary material for: CD4 T cell contact drives macrophage cell cycle G0-G1 transition
Source: Signal Transduct Target Ther. 2024 Dec 13;9:348. doi: 10.1038/s41392-024-02053-2 (PMC11638257; doi:10.1038/s41392-024-02053-2)
Supplement: Supplementary file 1 — supplementary material [file 41392_2024_2053_MOESM1_ESM.docx]

Supplementary Materials for

**CD4 T cell contact drives macrophage cell cycle G0-G1 transition**

Petra Mlcochova, Na Zhou, Omar Shabana, Roman Fischer, Ravindra K. Gupta^*^

*Correspondence to : Ravindra K Gupta; email [rkg20@cam.ac.uk](mailto:rkg20@cam.ac.uk)

**This file includes:**

Materials and Methods

**Materials and Methods**

**Cells, plasmids and viruses:**293T (a human embryonic kidney cell line, ATCC CRL‐3216) cells were maintained in Dulbecco's modified Eagle medium (DMEM) supplemented with 10% foetal calf serum (FCS), 100 U ml^−1^ penicillin and 100 mg ml^−1^ streptomycin, and regularly tested and found to be mycoplasma free. THP-1 cells, a kind gift from G. Towers, maintained in RPMI supplemented with 10% FCS, 100 U ml^−1^ penicillin and 100 mg ml^−1^ streptomycin. pBOB‐EF1‐FastFUCCI‐Puro was a gift from Kevin Brindle & Duncan Jodrell (Addgene plasmid # 86849; <http://n2t.net/addgene:86849>; RRID: Addgene_86849). pEXN‐MNCX, CMVi, and pMD2.G. plasmids were was kind gift from G. Towers.

### **Reagents, inhibitors, antibodies**

All chemicals were purchased from Sigma unless indicated otherwise. Kinase inhibitors used: CDK4/6 inhibitor (PD 0332991, Palbociclib) from Sigma; MEK/ERK inhibitor U0126 from Calbiochem (San Diego, USA); Antibodies used were as follows: anti‐cdc2 (Cell Signaling Technology, Beverly, MA, USA); anti‐SAMHD1 (ab67820, Abcam, UK), beta‐actin (ab6276, abcam, UK); mouse anti‐MCM2 (BM‐28, BD Biosciences, UK); and rabbit anti‐MCM2 (SP85) from Sigma; pSAMHD1 ProSci (Poway, CA, USA); anti‐Geminin (NCL‐L‐Geminin, Leica); anti-Ki67 (ab15580, abcam); anti‐human CD32, CD68, CD163, CD80, CD86, CD209, HLA-DR (BD, 550586, 562117, 563887, 340294, 555666, 551545, 567054). Kinase inhibitors used are CDK4/6 inhibitor (PD 0332991, Palbociclib, Sigma); MEK/ERK inhibitor U0126 (Calbiochem); CDK1 inhibitor (RO-3306, Sigma). Anti‐rabbit IgG, HRP‐linked antibody (7074) was from Cell Signaling. Mouse IgG HRP‐linked whole Ab (NXA931V) from Sigma. Goat anti‐mouse IgG (H+L) cross‐adsorbed secondary antibody: Alexa 488 (A‐11001), Alexa 594 (A‐11032); goat anti‐rabbit IgG (H+L) cross‐adsorbed secondary antibody Alexa 405 (A‐48254).

**Monocyte isolation and differentiation and CD4 isolation**: PBMC were prepared from apheresis cones from NHS Blood Center Cambridge by density‐gradient centrifugation (Lymphoprep, Axis‐Shield, UK). PBMCs were isolated from previously collected, non-identifiable leukocyte cones supplied by NHSBT Non-Clinical Issue in accordance with the terms of donor consent and UK HRA regulations. MDM were prepared by adherence with washing of non‐adherent cells after 2 h, with subsequent maintenance of adherent cells in RPMI 1640 medium supplemented with 10% human serum or 10% foetal calf serum and MCSF (10 ng/ml) for 3 days and then differentiated for a further 4 days in RPMI 1640 medium supplemented with 10% human/foetal calf sera without M‐CSF. Human AB serum (Sigma) was used to prepare unstimulated cells or FCS (Biosera or Sigma) to prepare stimulated cells. For macrophage polarisation cells were exposed at day 6 post-isolation with only medium (M0), stimulated with LPS (5ng/ml) /IFNgamma (20ng/ml) (to induce M1 macrophages or IL4 (20ng/ml) /IL-13 (20ng/ml) to induce M2 polarised. Autologous PBMC were frozen in FCS and 10% DMSO. PBMC were thawed and activated with Il-2/PHA for 2 days. CD4+ve cells were isolated from PBMCs with a negative CD4+ T cell isolation kit (130-096-533, Miltenyi Biotec) 1day prior addition to macrophages. CD4+ T cell isolation kit enriches a viable population of CD4+ T-cells. Furthermore, CD4+ cells were counted using Trypan Blue before addition to macrophages, ensuring the viability of CD4+ T-cells at >96%.

#### **Flow cytometry:** 1x10^6^ cells were incubated with directly-conjugated antibodies recognising macrophage cell surface markers for 30 minutes on ice. 2 mL of cold PBS was used to wash the cells. Cells were centrifuged at 300 x g for 5 minutes at 2° to 8°C, supernatant removed and cells resuspended in 0.5 mL of 1% paraformaldehyde solution. Positive populations were identified using flow cytometry using LSRFortessa X‐20 (BD Biosciences, UK) and FlowJo software (Tree Star, OR, USA).

**SDS PAGE and immunoblot:** Cells were lysed in reducing Laemmli SDS sample buffer containing PhosSTOP (Phosphatase Inhibitor Cocktail Tablets, Roche, Switzerland) at 96°C for 10 min and the proteins separated on NuPAGE^®^ Novex^®^ 4–12% Bis–Tris Gels. Subsequently, the proteins were transferred onto PVDF membranes (Millipore, Billerica, MA, USA), the membranes were quenched and proteins were detected using specific antibodies. Labelled protein bands were detected using Amersham ECL Prime Western Blotting Detection Reagent (GE Healthcare, USA) and ChemiDoc MP Imaging System (Bio‐Rad) CCD camera. Protein band intensities were quantified using ChemiDoc MP Imaging System and Image Lab software (Bio‐Rad, Hercules, CA, USA).

**Immunofluorescence:** Cells were fixed in 4% PFA, quenched with 50 mM NH_4_Cl and permeabilised with 0.1% Triton X‐100 in PBS. After blocking in PBS/1% FCS, cells were labelled for 1 h with primary antibodies diluted in PBS/1% FCS, washed and labelled again with Alexa Fluor secondary antibodies for 1 h. Cells were washed in PBS/1% FCS and stained with DAPI in PBS for 5 min. Labelled cells were detected using ArrayScan high‐content system (Thermo Fisher, Waltham, MA, USA) and analysed using Harmony (PerkinElmer, Waltham, MA, USA) and ImageJ software.

**VSV-G pseudotyped virus single-round infection**: VSV-G HIV-1 GFP virus was produced by transfection of 293T with GFP-encoding genome CSGW, packaging plasmid p8.91 and pMDG. VSV‐G‐pseudotyped HIV‐1 GFP expressing viruses were added to MDM. 4h post-incubation, the inoculum was removed and cells were washed once in a culture medium. This was left for 36 hr post infection before the cells were stained by Hoechst for nuclei. The percentage of infected GFP-expressing cells versus total cells was determined 48 h post‐infection using ArrayScan high‐content system (Thermo Fisher, Waltham, MA, USA) and analysed using Harmony and ImageJ software.

**Proteomics:** Phosphoproteins of donor cells were enriched with the Phosphoprotein Enrichment Kit (Thermo Pierce) according to manufacturer’s instructions and subsequently prepared in parallel for analysis by LC-MS/MS in parallel with not enriched lysates.Briefly, samples were reduced with DTT (5mM, 30 minutes at room temperature), alkylated with Iodoacetamide (20mM, 30 minutes in the dark), subjected to protein precipitation using Chloroform/Methanol and digested with trypsin (Promega). Desalted and acidified peptides (0.1% TFA, SOLA (Thermo Pierce)) were injected into a LC-MS/MS platform consisting of Q-Exactive mass spectrometer and Dionex Ultimate 3000 UPLC, after separation on a EASY Spray column (50cm, all Thermo) with a gradient of 2-35% acetonitrile in 0.1% formic acid/5% DMSO over 60 minutes. Data were acquired in DDA mode using standard parameters and imported into Progenesis QI (Waters) for label free quantitation (identification through Mascot 2.5 (Matrix Science)). Peptides failing the 1% FDR threshold and with a Mascot score <20 were excluded from analysis. The mass spectrometry proteomics data have been deposited to the ProteomeXchange Consortium via the PRIDE partner repository (https://www.ebi.ac.uk/pride/) with the dataset identifier PXD048462 and 10.6019/PXD048462.

**Phosphoproteomic bioinformatic analyses:** We first analysed phosphoproteins using the "DEP" R package. We compared the differential expression of phosphoproteins in times 0 vs 5, 5 vs 120 and 0 vs 120, and the threshold of DE is log2foldchange = 1 and p-value < 0.05. DEP: [https://rdrr.io/bioc/DEP/man/DEP.html](https://eur03.safelinks.protection.outlook.com/?url=https%3A%2F%2Frdrr.io%2Fbioc%2FDEP%2Fman%2FDEP.html&data=05%7C01%7Crkg20%40universityofcambridgecloud.onmicrosoft.com%7C75de54dfdfdb45c0c4c308db75d364af%7C49a50445bdfa4b79ade3547b4f3986e9%7C1%7C0%7C638233324629214082%7CUnknown%7CTWFpbGZsb3d8eyJWIjoiMC4wLjAwMDAiLCJQIjoiV2luMzIiLCJBTiI6Ik1haWwiLCJXVCI6Mn0%3D%7C3000%7C%7C%7C&sdata=AW%2B6eJtUR1W%2BdonGre1kTkrJDzNAX1XqoJPkzREX5wE%3D&reserved=0). We performed KEGG pathway enrichment analysis with pathfindR, using the default settings. Greedy algorithm for active subnetwork search was used, and the analysis was carried out over 10 iterations. The enrichment significance cutoff value was set to p < 0.05 for each analysis.
